# 2-[2-(4-Meth­oxy­phen­yl)-4,5-diphenyl-1*H*-imidazol-1-yl]ethanol

**DOI:** 10.1107/S1600536813004285

**Published:** 2013-03-02

**Authors:** Shaaban Kamel Mohamed, Mehmet Akkurt, Adel A. Marzouk, Vagif. M. Abbasov, Atash V. Gurbanov

**Affiliations:** a Chemistry and Environmental Division, Manchester Metropolitan University, Manchester M1 5GD, England; b Chemistry Department, Faculty of Science, Minia University, 61519 El-Minia, Egypt; c Department of Physics, Faculty of Sciences, Erciyes University, 38039 Kayseri, Turkey; d Pharmaceutical Chemistry Department, Faculty of Pharmacy, Al Azhar University, Egypt; e Mamedaliev Institute of Petrochemical Processes, National Academy of Sciences of Azerbaijan, Baku, Azerbaijan; f Department of Organic Chemistry, Baku State University, Baku, Azerbaijan

## Abstract

In the title compound, C_24_H_22_N_2_O_2_, the central imidazole ring makes dihedral angles of 49.45 (8), 88.94 (9) and 19.43 (8)° with the benzene ring and the two phenyl rings, respectively. The dihedral angle between the phenyl rings is 77.86 (9)°, and they form dihedral angles of 49.06 (9) and 67.31 (8)° with the benzene ring. In the crystal, mol­ecules are linked by O—H⋯N hydrogen bonds, forming chains along the *b* axis. These chains are connected by C—H⋯O hydrogen bonds, forming a two-dimensional network parallel to (100). In addition, C—H⋯π inter­actions are also observed. The terminal C and O atoms of the ethanol group are disordered over two sets of sites with an occupancy ratio of 0.801 (5):0.199 (5).

## Related literature

For imidazole derivatives as anti­cancer agents, see, for example: Krezel (1998 ▶); Andreani *et al.* (2000 ▶). For related structures, see: Akkurt *et al.* (2012 ▶); Mohamed *et al.* (2012 ▶). For further biological applications of imidazoles, see: Maier *et al.* (1989*a* ▶,*b* ▶). For standard bond-length data, see: Allen *et al.* (1987 ▶).
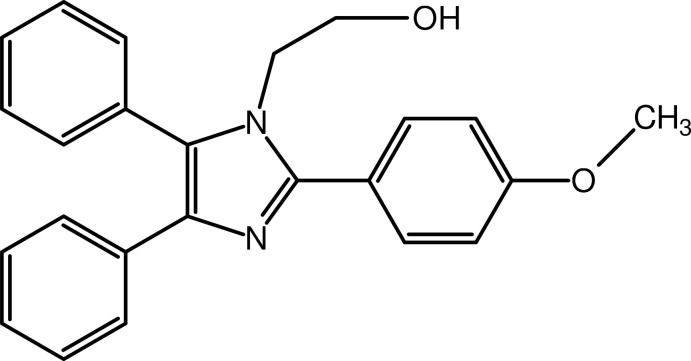


## Experimental

### 

#### Crystal data

C_24_H_22_N_2_O_2_
*M* *_r_* = 370.44
Monoclinic, 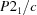

*a* = 14.3570 (4) Å
*b* = 13.2820 (4) Å
*c* = 10.7380 (3) Å
β = 108.212 (1)°
*V* = 1945.05 (10) Å^3^
*Z* = 4
Mo *K*α radiation
μ = 0.08 mm^−1^
*T* = 296 K
0.30 × 0.30 × 0.30 mm

#### Data collection

Bruker APEXII CCD diffractometer
Absorption correction: multi-scan (*SADABS*; Sheldrick, 1996 ▶) *T*_min_ = 0.976, *T*_max_ = 0.976
18622 measured reflections
3822 independent reflections
3046 reflections with *I* > 2σ(*I*)
*R*_int_ = 0.022

#### Refinement

*R*[*F* ^2^ > 2σ(*F* ^2^)] = 0.043
*wR*(*F* ^2^) = 0.110
*S* = 1.04
3822 reflections
269 parameters
2 restraints
H-atom parameters constrained
Δρ_max_ = 0.16 e Å^−3^
Δρ_min_ = −0.18 e Å^−3^

### 

Data collection: *APEX2* (Bruker, 2005 ▶); cell refinement: *SAINT-Plus* (Bruker, 2001 ▶); data reduction: *SAINT-Plus*; program(s) used to solve structure: *SIR97* (Altomare *et al.*, 1999 ▶); program(s) used to refine structure: *SHELXL97* (Sheldrick, 2008 ▶); molecular graphics: *ORTEP-3 for Windows* (Farrugia, 2012 ▶); software used to prepare material for publication: *WinGX* (Farrugia, 2012 ▶) and *PLATON* (Spek, 2009 ▶).

## Supplementary Material

Crystal structure: contains datablock(s) global, I. DOI: 10.1107/S1600536813004285/su2561sup1.cif

Structure factors: contains datablock(s) I. DOI: 10.1107/S1600536813004285/su2561Isup2.hkl

Supplementary material file. DOI: 10.1107/S1600536813004285/su2561Isup3.cml

Additional supplementary materials:  crystallographic information; 3D view; checkCIF report

## Figures and Tables

**Table 1:** Hydrogen-bond geometry (Å, °) *Cg*1 is the centroid of the C4–C9 benzene ring.

*D*—H⋯*A*	*D*—H	H⋯*A*	*D*⋯*A*	*D*—H⋯*A*
O1*A*—H1*OA*⋯N2^i^	0.82	2.01	2.829 (3)	175
C9—H9⋯O1*A* ^ii^	0.93	2.58	3.452 (3)	156
C24—H24⋯O1*A* ^iii^	0.93	2.53	3.448 (4)	170
C23—H23⋯*Cg*1^iii^	0.93	2.90	3.736 (2)	151

Symmetry codes: (i) 
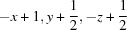
; (ii) 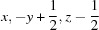; (iii) 
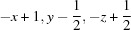
.

**Fig. 1.**
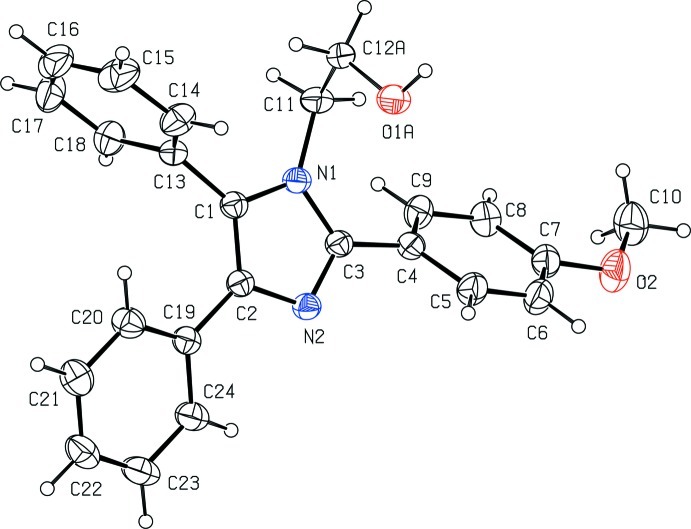
A view of the molecular structure of the title compound, with atom labelling. Displacement ellipsoids are drawn at the 30% probability level.

**Fig. 2.**
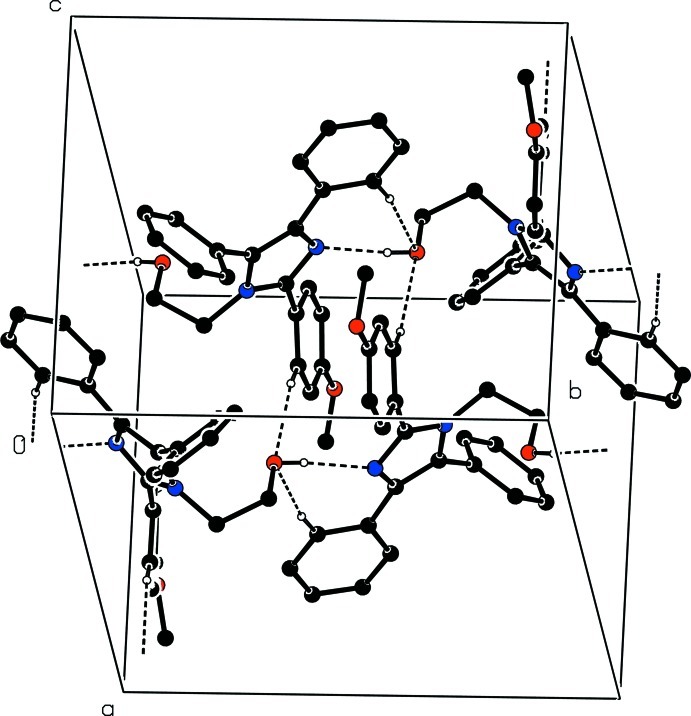
A partial view of the crystal packing of the title compound, showing the O—H···N and C—H···O hydrogen bonds (dashed lines; see Table 1 for details). H atoms not involved in hydrogen bonding have been omitted for clarity.

## supplementary crystallographic information

### Comment

Heterocyclic compounds such as imidazoles have been a traditional focal point for the development of new bio-active molecules such as anticancer agents (Krezel, 1998; Andreani *et al.* 2000). Many of the substituted imidazoles are known as inhibitors of fungicides and herbicides, plant growth regulators and therapeutic agents (Maier *et al.* 1989**a*,b*). As part of our on-going study to develop new routes for synthesis of tetra-substituted imidazole based amino alcohol compounds, we herein report the synthesis and crystal structure of the title compound.

In the title compound, Fig. 1, the central 1*H*-imidazole ring (N1/N2/C1—C3) makes dihedral angles of 49.45 (8), 88.94 (9) and 19.43 (8)°, with the benzene ring (C4–C9) and two phenyl rings (C13–C18 and C19–C24), respectively. The dihedral angle between the (C13–C18 and C19–C24) phenyl rings is 77.86 (9)°. The (C4–C9) benzene ring forms dihedral angles of 49.06 (9) and 67.31 (8)° with two the phenyl rings (C13–C18 and C19–C24), respectively. The N1–C11–C12A–O1A torsion angle is 46.6 (2)°. The values of the bond lengths (Allen *et al.*, 1987) and angles are within normal ranges and are comparable to those reported for related structures (Akkurt *et al.*, 2012; Mohamed *et al.*, 2012).

In the crystal, O—H···N hydrogen bonds (Table 1 and Fig 2) connect the molecules to form chains along the *b* axis direction. These chains are linked by C—H···O hydrogen bonds forming a two-dimensional network parallel to the bc plane. There are also C—H···π interactions present (Table 1).

### Experimental

A mixture of 2.1 g (10 mmol) 1,2-diphenylethane-1,2-dione, 1.36 g (10 mmol) 4-methoxybenzaldehyde, 0.67 g (11 mmol) 2-aminoethanol and 0.77 g (10 mmol) ammonium acetate was added to 0.5 g (3 mmol) of a fresh prepared diethyl ammonium hydrogen sulfate as an ionic liquid. The reaction mixture was heated on oil bath at 373 K and monitored by TLC till completion after 30 min then poured on water. The obtained solid was filtered off, washed with cold ethanol and dried under vacuum. The crude product was crystallized from ethanol to afford colourless prisms (m.p. 460 – 462 K), by slow evaporation at room temperature, in excellent yield (95%).

### Refinement

H atoms were positioned geometrically, with O—H = 0.82 Å, C—H = 0.93 Å (aromatic), 0.97 Å (methylene) and 0.98 Å (methine) H atoms, respectively, and refined as riding with *U*_iso_(H) = 1.5 *U*_eq_(O) for hydroxyl H atoms, and = 1.2 *U*_eq_(C) for other H atoms. Atoms C12 and O1 of the ethanol group are disordered over two sites (A and B), with occupancies of 0.801 (5):0.199 (5). Atoms O1A and O1B were refined with the EADP command.

### Crystal data

**Table:**

C_24_H_22_N_2_O_2_	*F*(000) = 784
*M**_r_* = 370.44	*D*_x_ = 1.265 Mg m^−^^3^
Monoclinic, *P*2_1_/*c*	Mo *K*α radiation, λ = 0.71073 Å
Hall symbol: -P 2ybc	Cell parameters from 6445 reflections
*a* = 14.3570 (4) Å	θ = 2.5–28.1°
*b* = 13.2820 (4) Å	µ = 0.08 mm^−^^1^
*c* = 10.7380 (3) Å	*T* = 296 K
β = 108.212 (1)°	Prism, colourless
*V* = 1945.05 (10) Å^3^	0.30 × 0.30 × 0.30 mm
*Z* = 4	

### Data collection

**Table:**

Bruker APEXII CCD diffractometer	3822 independent reflections
Radiation source: fine-focus sealed tube	3046 reflections with *I* > 2σ(*I*)
Graphite monochromator	*R*_int_ = 0.022
φ and ω scans	θ_max_ = 26.0°, θ_min_ = 1.5°
Absorption correction: multi-scan (*SADABS*; Sheldrick, 1996)	*h* = −17→17
*T*_min_ = 0.976, *T*_max_ = 0.976	*k* = −16→16
18622 measured reflections	*l* = −13→13

### Refinement

**Table:**

Refinement on *F*^2^	Primary atom site location: structure-invariant direct methods
Least-squares matrix: full	Secondary atom site location: difference Fourier map
*R*[*F*^2^ > 2σ(*F*^2^)] = 0.043	Hydrogen site location: inferred from neighbouring sites
*wR*(*F*^2^) = 0.110	H-atom parameters constrained
*S* = 1.04	*w* = 1/[σ^2^(*F*_o_^2^) + (0.0503*P*)^2^ + 0.4175*P*] where *P* = (*F*_o_^2^ + 2*F*_c_^2^)/3
3822 reflections	(Δ/σ)_max_ < 0.001
269 parameters	Δρ_max_ = 0.16 e Å^−^^3^
2 restraints	Δρ_min_ = −0.18 e Å^−^^3^

### Special details

**Table:**

Geometry. Bond distances, angles *etc*. have been calculated using the rounded fractional coordinates. All su's are estimated from the variances of the (full) variance-covariance matrix. The cell e.s.d.'s are taken into account in the estimation of distances, angles and torsion angles
Refinement. Refinement on *F*^2^ for ALL reflections except those flagged by the user for potential systematic errors. Weighted *R*-factors *wR* and all goodnesses of fit *S* are based on *F*^2^, conventional *R*-factors *R* are based on *F*, with *F* set to zero for negative *F*^2^. The observed criterion of *F*^2^ > σ(*F*^2^) is used only for calculating -*R*-factor-obs *etc*. and is not relevant to the choice of reflections for refinement. *R*-factors based on *F*^2^ are statistically about twice as large as those based on *F*, and *R*-factors based on ALL data will be even larger.

### Fractional atomic coordinates and isotropic or equivalent isotropic displacement parameters (Å^2^)

**Table:**

	*x*	*y*	*z*	*U*_iso_*/*U*_eq_	Occ. (<1)
O1A	0.4684 (2)	0.37249 (19)	0.2105 (2)	0.0601 (7)	0.801 (5)
O2	0.85914 (8)	0.08118 (11)	0.17179 (13)	0.0735 (5)	
N1	0.39627 (8)	0.18785 (8)	0.09335 (11)	0.0404 (3)	
N2	0.43176 (8)	0.05860 (9)	0.22977 (11)	0.0417 (4)	
C1	0.31198 (10)	0.16540 (10)	0.12402 (13)	0.0392 (4)	
C2	0.33467 (10)	0.08474 (10)	0.20781 (13)	0.0386 (4)	
C3	0.46645 (10)	0.12152 (10)	0.16028 (14)	0.0400 (4)	
C4	0.56824 (10)	0.11574 (11)	0.15705 (14)	0.0426 (4)	
C5	0.64400 (12)	0.10619 (14)	0.27435 (16)	0.0554 (6)	
C6	0.73926 (12)	0.09378 (15)	0.27566 (17)	0.0621 (6)	
C7	0.76170 (11)	0.09152 (12)	0.16002 (17)	0.0523 (5)	
C8	0.68762 (12)	0.09900 (12)	0.04244 (16)	0.0518 (5)	
C9	0.59175 (11)	0.11052 (12)	0.04197 (15)	0.0496 (5)	
C10	0.88821 (14)	0.09847 (18)	0.0591 (2)	0.0797 (8)	
C11	0.40902 (12)	0.27268 (11)	0.01273 (15)	0.0507 (5)	
C12A	0.40758 (15)	0.37524 (14)	0.0781 (2)	0.0462 (7)	0.801 (5)
C13	0.21836 (10)	0.22019 (11)	0.06779 (14)	0.0428 (5)	
C14	0.19561 (13)	0.30302 (13)	0.13025 (18)	0.0605 (6)	
C15	0.10593 (15)	0.34991 (17)	0.0819 (2)	0.0764 (8)	
C16	0.03897 (14)	0.31534 (19)	−0.0294 (2)	0.0799 (8)	
C17	0.06034 (14)	0.23439 (19)	−0.0938 (2)	0.0808 (8)	
C18	0.15028 (12)	0.18658 (14)	−0.04567 (17)	0.0616 (6)	
C19	0.27150 (10)	0.02319 (10)	0.26213 (13)	0.0413 (4)	
C20	0.18141 (12)	0.05525 (13)	0.26891 (17)	0.0543 (6)	
C21	0.12225 (13)	−0.00802 (14)	0.31328 (18)	0.0628 (6)	
C22	0.15231 (14)	−0.10348 (14)	0.35442 (18)	0.0632 (7)	
C23	0.24208 (15)	−0.13547 (14)	0.3522 (2)	0.0707 (7)	
C24	0.30092 (13)	−0.07337 (12)	0.30620 (18)	0.0593 (6)	
O1B	0.4482 (10)	0.3797 (10)	0.1690 (11)	0.0601 (7)	0.199 (5)
C12B	0.4757 (6)	0.3448 (6)	0.0623 (8)	0.053 (3)	0.199 (5)
H6	0.78890	0.08690	0.35510	0.0750*	
H8	0.70200	0.09630	−0.03620	0.0620*	
H9	0.54190	0.11480	−0.03780	0.0600*	
H10A	0.86270	0.04600	−0.00370	0.1200*	
H10B	0.95850	0.09890	0.08350	0.1200*	
H1OA	0.49500	0.42730	0.23080	0.0900*	0.801 (5)
H5	0.63000	0.10820	0.35320	0.0670*	
H11B	0.47090	0.26510	−0.00480	0.0610*	0.801 (5)
H12A	0.43070	0.42700	0.03120	0.0550*	0.801 (5)
H12B	0.34100	0.39170	0.07450	0.0550*	0.801 (5)
H14	0.24140	0.32750	0.20600	0.0730*	
H15	0.09120	0.40520	0.12540	0.0920*	
H16	−0.02160	0.34690	−0.06170	0.0960*	
H17	0.01440	0.21120	−0.17030	0.0970*	
H18	0.16470	0.13160	−0.09010	0.0740*	
H20	0.16040	0.12040	0.24320	0.0650*	
H21	0.06140	0.01450	0.31520	0.0750*	
H22	0.11210	−0.14610	0.38360	0.0760*	
H23	0.26370	−0.19970	0.38190	0.0850*	
H24	0.36160	−0.09670	0.30470	0.0710*	
H10C	0.86310	0.16230	0.02120	0.1200*	
H11A	0.35710	0.27100	−0.07060	0.0610*	0.801 (5)
H1OB	0.47760	0.43200	0.19690	0.0900*	0.199 (5)
H11C	0.42580	0.24450	−0.06080	0.0610*	0.199 (5)
H11D	0.34540	0.30450	−0.02310	0.0610*	0.199 (5)
H12C	0.47170	0.39800	−0.00110	0.0640*	0.199 (5)
H12D	0.54170	0.31760	0.09060	0.0640*	0.199 (5)

### Atomic displacement parameters (Å^2^)

**Table:**

	*U*^11^	*U*^22^	*U*^33^	*U*^12^	*U*^13^	*U*^23^
O1A	0.0760 (15)	0.0472 (8)	0.0422 (14)	−0.0150 (9)	−0.0031 (11)	0.0009 (10)
O2	0.0444 (6)	0.1058 (11)	0.0707 (8)	0.0008 (6)	0.0188 (6)	−0.0010 (7)
N1	0.0451 (6)	0.0354 (6)	0.0391 (6)	−0.0005 (5)	0.0107 (5)	0.0024 (5)
N2	0.0440 (7)	0.0361 (6)	0.0427 (7)	0.0011 (5)	0.0102 (5)	0.0016 (5)
C1	0.0428 (7)	0.0357 (7)	0.0378 (7)	−0.0013 (6)	0.0108 (6)	−0.0019 (6)
C2	0.0425 (7)	0.0345 (7)	0.0369 (7)	−0.0003 (6)	0.0099 (6)	−0.0029 (6)
C3	0.0437 (8)	0.0353 (7)	0.0384 (7)	−0.0007 (6)	0.0091 (6)	−0.0010 (6)
C4	0.0434 (8)	0.0378 (7)	0.0445 (8)	−0.0017 (6)	0.0106 (6)	−0.0002 (6)
C5	0.0494 (9)	0.0732 (11)	0.0426 (9)	−0.0019 (8)	0.0128 (7)	0.0073 (8)
C6	0.0453 (9)	0.0859 (13)	0.0487 (10)	−0.0025 (8)	0.0054 (7)	0.0085 (9)
C7	0.0424 (8)	0.0537 (9)	0.0595 (10)	−0.0027 (7)	0.0141 (7)	−0.0029 (7)
C8	0.0532 (9)	0.0559 (9)	0.0478 (9)	−0.0034 (7)	0.0179 (7)	−0.0124 (7)
C9	0.0471 (8)	0.0538 (9)	0.0434 (8)	−0.0008 (7)	0.0077 (7)	−0.0101 (7)
C10	0.0578 (11)	0.1018 (16)	0.0886 (15)	−0.0032 (10)	0.0361 (11)	−0.0036 (12)
C11	0.0593 (9)	0.0455 (9)	0.0474 (9)	0.0017 (7)	0.0170 (7)	0.0112 (7)
C12A	0.0448 (12)	0.0388 (10)	0.0489 (13)	−0.0008 (8)	0.0059 (10)	0.0084 (8)
C13	0.0429 (8)	0.0424 (8)	0.0421 (8)	−0.0005 (6)	0.0118 (6)	0.0086 (6)
C14	0.0619 (10)	0.0560 (10)	0.0594 (10)	0.0146 (8)	0.0130 (8)	0.0008 (8)
C15	0.0730 (13)	0.0769 (13)	0.0854 (15)	0.0310 (11)	0.0337 (12)	0.0189 (11)
C16	0.0457 (10)	0.1047 (17)	0.0919 (16)	0.0193 (11)	0.0254 (11)	0.0434 (14)
C17	0.0498 (11)	0.1072 (17)	0.0695 (13)	−0.0099 (11)	−0.0044 (9)	0.0164 (12)
C18	0.0567 (10)	0.0660 (11)	0.0535 (10)	−0.0065 (8)	0.0049 (8)	0.0003 (8)
C19	0.0475 (8)	0.0397 (8)	0.0350 (7)	−0.0050 (6)	0.0105 (6)	−0.0034 (6)
C20	0.0587 (10)	0.0475 (9)	0.0615 (10)	0.0017 (7)	0.0259 (8)	0.0015 (8)
C21	0.0595 (10)	0.0703 (12)	0.0669 (11)	−0.0057 (9)	0.0317 (9)	−0.0016 (9)
C22	0.0722 (12)	0.0628 (11)	0.0594 (11)	−0.0201 (9)	0.0277 (9)	0.0015 (9)
C23	0.0804 (13)	0.0481 (10)	0.0867 (14)	−0.0044 (9)	0.0308 (11)	0.0179 (9)
C24	0.0584 (10)	0.0467 (9)	0.0751 (12)	0.0015 (8)	0.0244 (9)	0.0127 (8)
O1B	0.0760 (15)	0.0472 (8)	0.0422 (14)	−0.0150 (9)	−0.0031 (11)	0.0009 (10)
C12B	0.051 (5)	0.053 (5)	0.058 (5)	−0.005 (4)	0.021 (4)	−0.007 (4)

### Geometric parameters (Å, º)

**Table:**

O1A—C12A	1.417 (3)	C19—C24	1.386 (2)
O1B—C12B	1.402 (15)	C20—C21	1.381 (3)
O2—C10	1.417 (2)	C21—C22	1.367 (3)
O2—C7	1.372 (2)	C22—C23	1.364 (3)
O1A—H1OA	0.8200	C23—C24	1.378 (3)
O1B—H1OB	0.8200	C5—H5	0.9300
N1—C11	1.4668 (19)	C6—H6	0.9300
N1—C1	1.3824 (19)	C8—H8	0.9300
N1—C3	1.3621 (18)	C9—H9	0.9300
N2—C2	1.3834 (19)	C10—H10A	0.9600
N2—C3	1.3174 (18)	C10—H10C	0.9600
C1—C2	1.3712 (19)	C10—H10B	0.9600
C1—C13	1.481 (2)	C11—H11A	0.9700
C2—C19	1.470 (2)	C11—H11C	0.9700
C3—C4	1.475 (2)	C11—H11D	0.9700
C4—C9	1.381 (2)	C11—H11B	0.9700
C4—C5	1.389 (2)	C12A—H12A	0.9700
C5—C6	1.373 (3)	C12A—H12B	0.9700
C6—C7	1.377 (2)	C12B—H12C	0.9700
C7—C8	1.377 (2)	C12B—H12D	0.9700
C8—C9	1.383 (2)	C14—H14	0.9300
C11—C12B	1.341 (9)	C15—H15	0.9300
C11—C12A	1.536 (2)	C16—H16	0.9300
C13—C14	1.380 (2)	C17—H17	0.9300
C13—C18	1.377 (2)	C18—H18	0.9300
C14—C15	1.377 (3)	C20—H20	0.9300
C15—C16	1.358 (3)	C21—H21	0.9300
C16—C17	1.364 (3)	C22—H22	0.9300
C17—C18	1.386 (3)	C23—H23	0.9300
C19—C20	1.385 (2)	C24—H24	0.9300
			
C7—O2—C10	117.99 (15)	C9—C8—H8	120.00
C12A—O1A—H1OA	109.00	C7—C8—H8	120.00
C12B—O1B—H1OB	110.00	C4—C9—H9	119.00
C1—N1—C11	125.90 (12)	C8—C9—H9	119.00
C3—N1—C11	126.88 (13)	O2—C10—H10B	109.00
C1—N1—C3	107.04 (11)	O2—C10—H10C	109.00
C2—N2—C3	106.46 (12)	O2—C10—H10A	109.00
N1—C1—C2	106.18 (12)	H10A—C10—H10C	109.00
C2—C1—C13	130.59 (14)	H10B—C10—H10C	109.00
N1—C1—C13	123.19 (12)	H10A—C10—H10B	110.00
N2—C2—C1	109.15 (13)	N1—C11—H11A	109.00
C1—C2—C19	130.18 (14)	N1—C11—H11C	107.00
N2—C2—C19	120.43 (12)	N1—C11—H11D	107.00
N1—C3—N2	111.16 (13)	N1—C11—H11B	109.00
N1—C3—C4	126.61 (13)	C12A—C11—H11B	109.00
N2—C3—C4	122.22 (13)	H11A—C11—H11B	108.00
C3—C4—C9	123.02 (13)	C12B—C11—H11C	106.00
C5—C4—C9	117.76 (15)	C12B—C11—H11D	108.00
C3—C4—C5	118.97 (14)	H11C—C11—H11D	107.00
C4—C5—C6	121.02 (15)	C12A—C11—H11A	109.00
C5—C6—C7	120.42 (16)	O1A—C12A—H12B	110.00
O2—C7—C8	124.40 (16)	C11—C12A—H12A	110.00
O2—C7—C6	116.00 (15)	O1A—C12A—H12A	110.00
C6—C7—C8	119.60 (16)	H12A—C12A—H12B	108.00
C7—C8—C9	119.60 (15)	C11—C12A—H12B	110.00
C4—C9—C8	121.57 (15)	C11—C12B—H12D	111.00
N1—C11—C12B	121.3 (4)	O1B—C12B—H12C	111.00
N1—C11—C12A	112.93 (13)	H12C—C12B—H12D	109.00
O1A—C12A—C11	110.10 (17)	O1B—C12B—H12D	111.00
O1B—C12B—C11	102.2 (8)	C11—C12B—H12C	111.00
C1—C13—C14	121.08 (14)	C13—C14—H14	120.00
C14—C13—C18	118.58 (15)	C15—C14—H14	120.00
C1—C13—C18	120.29 (14)	C16—C15—H15	120.00
C13—C14—C15	120.84 (17)	C14—C15—H15	120.00
C14—C15—C16	120.0 (2)	C15—C16—H16	120.00
C15—C16—C17	120.2 (2)	C17—C16—H16	120.00
C16—C17—C18	120.20 (19)	C16—C17—H17	120.00
C13—C18—C17	120.17 (17)	C18—C17—H17	120.00
C2—C19—C20	123.49 (13)	C17—C18—H18	120.00
C2—C19—C24	119.33 (14)	C13—C18—H18	120.00
C20—C19—C24	117.15 (15)	C19—C20—H20	119.00
C19—C20—C21	121.07 (16)	C21—C20—H20	119.00
C20—C21—C22	120.64 (18)	C20—C21—H21	120.00
C21—C22—C23	119.18 (18)	C22—C21—H21	120.00
C22—C23—C24	120.54 (18)	C23—C22—H22	120.00
C19—C24—C23	121.37 (17)	C21—C22—H22	120.00
C6—C5—H5	119.00	C22—C23—H23	120.00
C4—C5—H5	119.00	C24—C23—H23	120.00
C5—C6—H6	120.00	C23—C24—H24	119.00
C7—C6—H6	120.00	C19—C24—H24	119.00
			
C10—O2—C7—C6	−167.65 (17)	N2—C3—C4—C5	−47.3 (2)
C10—O2—C7—C8	12.7 (3)	N2—C3—C4—C9	126.90 (16)
C3—N1—C1—C2	−0.76 (14)	C3—C4—C5—C6	175.61 (16)
C3—N1—C1—C13	−178.72 (13)	C9—C4—C5—C6	1.1 (3)
C11—N1—C1—C2	−176.05 (12)	C3—C4—C9—C8	−176.00 (14)
C11—N1—C1—C13	6.0 (2)	C5—C4—C9—C8	−1.8 (2)
C1—N1—C3—N2	0.55 (15)	C4—C5—C6—C7	0.6 (3)
C1—N1—C3—C4	179.26 (13)	C5—C6—C7—O2	178.52 (17)
C11—N1—C3—N2	175.78 (12)	C5—C6—C7—C8	−1.8 (3)
C11—N1—C3—C4	−5.5 (2)	O2—C7—C8—C9	−179.18 (15)
C1—N1—C11—C12A	67.64 (19)	C6—C7—C8—C9	1.2 (2)
C3—N1—C11—C12A	−106.74 (18)	C7—C8—C9—C4	0.6 (2)
C3—N2—C2—C1	−0.39 (15)	N1—C11—C12A—O1A	46.6 (2)
C3—N2—C2—C19	174.53 (12)	C1—C13—C14—C15	−175.92 (17)
C2—N2—C3—N1	−0.10 (16)	C18—C13—C14—C15	1.4 (3)
C2—N2—C3—C4	−178.89 (13)	C1—C13—C18—C17	176.12 (17)
N1—C1—C2—N2	0.71 (15)	C14—C13—C18—C17	−1.2 (3)
N1—C1—C2—C19	−173.56 (13)	C13—C14—C15—C16	−0.7 (3)
C13—C1—C2—N2	178.46 (13)	C14—C15—C16—C17	−0.2 (3)
C13—C1—C2—C19	4.2 (2)	C15—C16—C17—C18	0.4 (3)
N1—C1—C13—C14	−91.53 (18)	C16—C17—C18—C13	0.4 (3)
N1—C1—C13—C18	91.21 (18)	C2—C19—C20—C21	175.87 (15)
C2—C1—C13—C14	91.1 (2)	C24—C19—C20—C21	−2.3 (2)
C2—C1—C13—C18	−86.2 (2)	C2—C19—C24—C23	−176.95 (16)
N2—C2—C19—C20	165.59 (14)	C20—C19—C24—C23	1.3 (2)
N2—C2—C19—C24	−16.3 (2)	C19—C20—C21—C22	1.4 (3)
C1—C2—C19—C20	−20.7 (2)	C20—C21—C22—C23	0.5 (3)
C1—C2—C19—C24	157.46 (15)	C21—C22—C23—C24	−1.5 (3)
N1—C3—C4—C5	134.15 (16)	C22—C23—C24—C19	0.6 (3)
N1—C3—C4—C9	−51.7 (2)		

### Hydrogen-bond geometry (Å, º)

Cg1 is the centroid of the C4–C9 benzene ring.

**Table:**

*D*—H···*A*	*D*—H	H···*A*	*D*···*A*	*D*—H···*A*
O1*A*—H1*OA*···N2^i^	0.82	2.01	2.829 (3)	175
C9—H9···O1*A*^ii^	0.93	2.58	3.452 (3)	156
C24—H24···O1*A*^iii^	0.93	2.53	3.448 (4)	170
C23—H23···*Cg*1^iii^	0.93	2.90	3.736 (2)	151

Symmetry codes: (i) −*x*+1, *y*+1/2, −*z*+1/2; (ii) *x*, −*y*+1/2, *z*−1/2; (iii) −*x*+1, *y*−1/2, −*z*+1/2.

## References

[bb1] Akkurt, M., Marzouk, A. A., Abbasov, V. M., Abdelhamid, A. A. & Gurbanov, A. V. (2012). *Acta Cryst.* E**68**, o3113–o3114. 10.1107/S1600536812041979 PMC3515219 23284439

[bb2] Allen, F. H., Kennard, O., Watson, D. G., Brammer, L., Orpen, A. G. & Taylor, R. (1987). *J. Chem. Soc. Perkin Trans. 2*, pp. S1–19.

[bb3] Altomare, A., Burla, M. C., Camalli, M., Cascarano, G. L., Giacovazzo, C., Guagliardi, A., Moliterni, A. G. G., Polidori, G. & Spagna, R. (1999). *J. Appl. Cryst.* **32**, 115–119.

[bb4] Andreani, A., Leoni, A., Locatelli, A., Morigi, R., Rambaldi, M., Recanatini, M. & Garaliene, V. (2000). *Bioorg. Med. Chem.* **8**, 2359–2366. 10.1016/s0968-0896(00)00165-6 11026549

[bb5] Bruker (2001). *SAINT-Plus* Bruker AXS Inc., Madison, Wisconsin, USA.

[bb6] Bruker (2005). *APEX2* Bruker AXS Inc., Madison, Wisconsin, USA.

[bb7] Farrugia, L. J. (2012). *J. Appl. Cryst.* **45**, 849–854.

[bb8] Krezel, I. (1998). *Il Farmaco*, **53**, 342–345. 10.1016/s0014-827x(98)00031-7 9679284

[bb9] Maier, T., Schmierer, R., Bauer, K., Bieringer, H., Buerstell, H. & Sachse, B. (1989*a*). US Patent 4 820 335.

[bb10] Maier, T., Schmierer, R., Bauer, K., Bieringer, H., Buerstell, H. & Sachse, B. (1989*b*). *Chem. Abstr.* **111**, 19494.

[bb11] Mohamed, S. K., Akkurt, M., Fronczek, F. R., Marzouk, A. A. E. & Abdelhamid, A. A. (2012). *Acta Cryst.* E**68**, o2979–o2980. 10.1107/S1600536812039566 PMC3470344 23125757

[bb12] Sheldrick, G. M. (1996). *SADABS* University of Göttingen, Germany.

[bb13] Sheldrick, G. M. (2008). *Acta Cryst.* A**64**, 112–122. 10.1107/S0108767307043930 18156677

[bb14] Spek, A. L. (2009). *Acta Cryst.* D**65**, 148–155. 10.1107/S090744490804362X PMC2631630 19171970

